# Primary Mechanism Study of *Panax notoginseng* Flower (Herb) on Myocardial Infarction in Rats

**DOI:** 10.1155/2019/8723076

**Published:** 2019-05-02

**Authors:** Xin Zhou, Zhi-Cheng Li, Pei-Pei Chen, Rui-Fang Xie

**Affiliations:** ^1^Longhua Hospital, Shanghai University of Traditional Chinese Medicine, Shanghai 200032, China; ^2^Shanghai Pu Dong Hospital, Fu Dan University, Shanghai 200433, China

## Abstract

**Background:**

*Panax notoginseng* (Burk.) F. H. Chen is one of the most common herbs in China. Because of its good efficacy and little adverse reaction, *Panax notoginseng* has been used widely to treat cardiovascular diseases (CVDs).

**Objective:**

To investigate effects of *Panax notoginseng* flower (PN-F) on rats with myocardial infarction (MI).

**Methods:**

The proximal left anterior descending coronary artery in rats was ligated to induce acute myocardial infarction. Then, animals were randomly assigned to four experimental groups: MI control group, Betaloc control group (with Betaloc 10 mg/kg/d), FD500 (low-dose) group (*Panax notoginseng* flower decoction 500 mg/kg, *n*=10), and FD1000 (high-dose) group (*Panax notoginseng* flower decoction 1000 mg/kg, *n*=10). *Panax notoginseng* flower decoction or Betaloc was orally administrated for two to four weeks before and after operation. Sham-operated group was used as a normal untreated group, in which animals were treated with double distilled water, once daily. HE (hematoxylin and eosin) staining, immunofluorescent assay, TUNEL assay, quantitative real-time PCR, and western blot analysis were, respectively, performed to observe morphology, count mean minimal vessels, investigate apoptotic cells, and record gene (HIF-1, VEGFA, and KDR) and protein (Bcl-2 and Bax) expressions.

**Results:**

Two weeks after MI, PN-F significantly enhanced capillary density in the border area of MI, decreased infarct size, improved minimal vessels, suppressed cell apoptosis, and enhanced expressions of genes (HIF-1, VEGFA, and KDR) and proteins (Bcl-2 and Bax).

**Conclusions:**

PN-F demonstrated a potential herb to treat rats with myocardial infarction through promoting angiogenesis and inhibition of apoptosis in the infarct area.

## 1. Introduction

Myocardial infarction is a major consequence of coronary artery disease, and heart remodeling secondary to myocardial infarction is considered the first step towards heart failure [1]. Both myocardial infarction and heart failure will result in high morbidity and mortality. At an early stage, myocardial ischemia could stimulate spontaneous angiogenesis to increase the perfusion of ischemic tissue and salvage ischemic myocardium [2]. However, physiological angiogenesis was slow (particularly in old individuals), and the number and size of new blood vessels were too small to sufficiently supply ischemic regions of the myocardium [3]. Hence, proangiogenic therapy, which could promote reperfusion and recover function of the ischemic heart, appeared to be a promising strategy. Some reports demonstrated that myocardial infarction was related to angiogenesis factors [4], including vascular endothelial growth factor-*α* (VEGFA), HIF-1 (hypoxia-inducible factor-1), and KDR (kinase insert domain receptor). Proangiogenesis improved the outcome of myocardial infarction [5]. However, clinical trials were not so exciting [6, 7]. The reasons might be lack of effective medicine.

*Panax notoginseng* (Burk.) F. H. Chen, also named Sanqi, is one of the most common herbs in China. According to TCM theory, it can dissipate blood stasis and arrest bleeding, remove swell, and relieve pain. Because of its good efficacy and little adverse reaction, *Panax notoginseng* has been used widely to treat cardiovascular diseases (CVDs) and has attracted more and more attention in many countries such as the United States, Japan, and Korea [8]. Usually, roots are considered an official part, which are listed in Chinese pharmacopeia. However, flowers of *Panax notoginseng* (PN-F) can also be used as folk herbs or diet supplements, which can treat hypertension, vertigo, and acute faucitis in China [9, 10]. PN-F contains plenty of saponins, including protopanaxadiol, protopanaxatriol, and triterpene saponins [11]. These saponins could attenuate inflammatory response [12], inhibit proliferation [13], and enhance both sperm motility and sperm progression [14]. More importantly, it was found PN-F had stronger angiogenesis effects on zebrafish than roots [15] and improved the ventricular hypertrophy state in human chymase transgenic mice through regulation of the expression of mRNA and protein of TGF-β/Smad in ventricular tissues [16]. Furthermore, our previous results demonstrated that the purified saponin preparation from PN-F could ameliorate acute myocardial infarction via proangiogenesis and antiapoptosis [17–19]. How about *Panax notoginseng* herb? Therefore, in the present study, we aimed to observe angiogenesis effects of *PN-F herb* on MI rats, figure out its possible mechanism, and provide a basis for the development of the proangiogenesis *herb*.

## 2. Materials and Methods

### 2.1. Medicinal Herb Extract

PN-F (Lot 120801) was bought from Tong-Ji-Tang Pharmaceutical Company in Shanghai, China. PN-F was extracted twice with 10-fold water at 100°C for 20 min. The extracts were filtrated, combined, and concentrated to 1 g rude herb per ml.

### 2.2. Content Analysis of Extract

The chromatographic system used is of Agilent 1200 HPLC series, consisting of a binary pump (Model G1312B), a vacuum degasser (Model G1379B), an autosampler (Model G1367C), and a column oven (Model G1316B). The mass spectrometer used was an Applied Biosystems Sciex 4000 Q-Trap® mass spectrometer (Foster, CA, USA) combined with a Source 5000 LC/MS gas generator (Parker Balston Analytical Gas Systems). Data acquisition was carried out by Analyst® 1.4.2 software on a Dell computer.

### 2.3. Animal Model

All experimental procedures were performed according to the guidelines of the National Animal Welfare Law of China (Ethical Approval No. SCXK 2012-00014). Seventy male Sprague-Dawley rats (100–110 g) were purchased from Shanghai Laboratory Animals Limited Liability Company (Shanghai, China). They were anesthetized with ether, and thoracotomy was performed through the fourth infarcted space [20, 21].

The rats were randomly assigned to four experimental groups: MI group (*n*=10), Betaloc group (Betaloc 1 mg/kg, *n*=10), FD500 group (*Panax notoginseng* flower decoction 500 mg/kg, *n*=10), and FD1000 group (*Panax notoginseng* flower decoction 1000 mg/kg, *n*=10). *Panax notoginseng* flower decoction or Betaloc was orally administrated once daily for two weeks before and after operation, totaling four weeks. In addition to the MI group, the sham-operated group (*n*=8) was also made with the same procedure but without the LAD ligation. Heart samples were collected at the 14^th^ day after MI.

**Figure 1:**
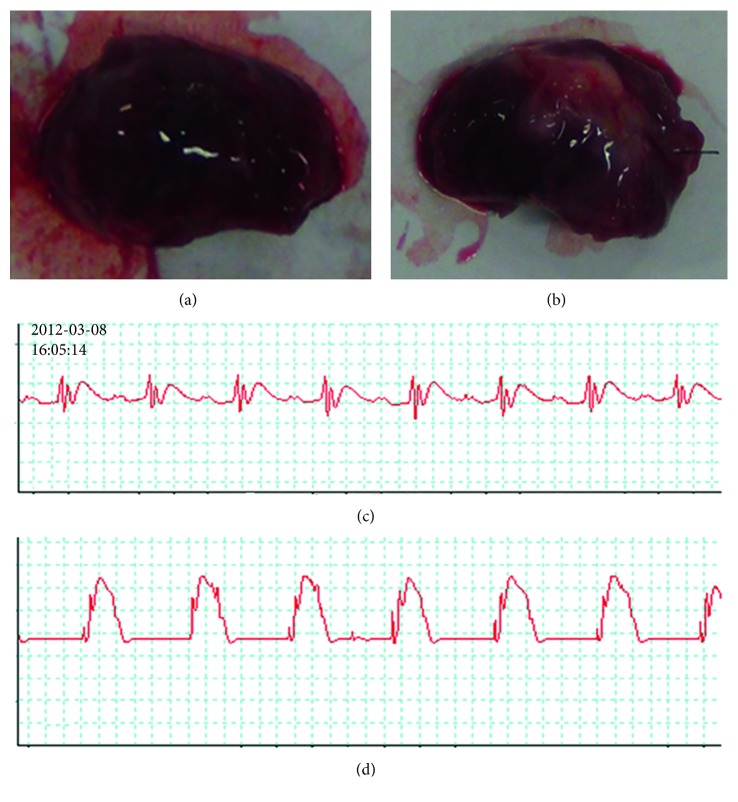
The LAD artery of rats was occluded to establish the MI control. Hearts of the sham (a) and MI (myocardial infarction) control (b) groups were quickly removed and observed. Electrocardiograms of rats in the sham (c) and MI control (d) groups were recorded. The arrow showed that the ST segment was elevated in myocardial infarcted animals.

The left anterior descending (LAD) coronary artery was identified and occluded with a 6-0 Prolene suture in the middle portion. The exposed heart was immediately pushed back into the thoracic cavity. The chest was closed to let the animals recover (Figure 1). Finally, antibiotics treatment was performed after the operation by applying penicillin powder to wounds in order to avoid infection.

### 2.4. Quantification of Myocardial Infarct Size and Histopathological Analysis

All the rats were sacrificed after 14 days, and the hearts were removed. The LV was cut into sections and mounted, and the infarct size was calculated as a percentage of the LV. The remaining rats' heart tissues were fixed in 10% formalin buffer for histopathological and immunofluorescent analysis. After fixing, the heart tissues were dehydrated with gradient ethanol, cleared, immersed, embedded in paraffin, and sliced to 5 *μ*m in thickness. These slices were then stained with hematoxylin and eosin (HE) for histopathologic observation.

### 2.5. Immunofluorescent Analysis

Paraffin slices were deparaffinized and rehydrated according to the standard protocol. Afterwards, these tissues needed antigen retrieval. In detail, dewaxed tissues were washed with PBS (phosphate-buffered saline) three times (3 min/time), soaked in 3% hydrogen peroxide for 10 min, and rinsed, respectively, with tap water for 30 s and PBS for 5 min. Then, the tissues were soaked in citrate antigen retrieval buffer, boiled over high heat for 8 min and medium heat for 5 min using a microwave, and cooled down under room temperature for 30 min. After three rounds of washing with PBS (3 min/time), the tissues were blocked with 5% goat serum (diluted with PBS) for 15 min under room temperature and then washed twice with PBS (3 min/time). The samples were incubated with VWF primary antibody (von Willebrand factor, Santa Cruz Biotechnology) and *α*-SMA antibody (*α*-Smooth Muscle Actin, Santa Cruz Biotechnology) for 2 h at room temperature. After washing with PBS three times (15 min/time), the samples were incubated with fluorescence-conjugated secondary antibody for 1 h at room temperature in dark place and washed with PBS three times again (10 min/time). Finally, the sections were dripped with the water-soluble DAPI (diamidino phenylindole) mounting medium, covered with a coverslip, sealed with nail polish, and stored at 4°C.

Under the fluorescence microscope of 400 magnified fields, the smooth muscle cells which expressed *α*-SMA antibody showed green fluorescence, while endothelial cells which expressed VWF antibody showed red fluorescence. Five zones of the peri-infarct zone and remote area were selected. The minimal vessels were counted and pictured. Average vessel numbers of the five zones were recorded as mean MVC (microvessel count).

### 2.6. TUNEL Assay

Paraffin slices were dewaxed and rehydrated. The antigen of tissues was repaired according to the above standard protocol. After washing with PBS, the slices were incubated with 0.1% TUNEL (terminal deoxynucleotidyl transferase-mediated dUTP nick-end labeling) antibody (In Situ Cell Death Detection Kit, Fluorescein, Roche; Lot #11684795910) overnight at 4°C. The slices were then washed twice with PBS (10 min/time), dripped with the DAPI mounting medium, covered with a coverslip, sealed with nail polish, and stored at 4°C. Under the fluorescence microscope of 200 magnified fields, apoptotic cells expressed green fluorescence, while the nucleus showed blue fluorescence. Five zones of the peri-infarct zone and remote area were selected. The apoptotic cells were counted. Average apoptotic cell numbers of the five zones were recorded.

### 2.7. Quantitative Real-Time PCR

Heart tissues were lysed with RNA lysis buffer containing 0.5% *ß*-mercaptoethanol on ice for 30 min. After centrifugation at 12,500*g* for 20 min at room temperature, the supernatant was collected. The total RNA concentration was quantified with a spectrophotometer. The total RNA (1 *μ*g) from each sample was reverse transcribed to cDNA in a 20 *μ*l reaction mixture. The obtained cDNA was diluted with 980 *μ*l cDNA buffer (1% 5X Phusion HF reaction buffer, Biolabs) to final solution (1 *μ*g/ml). Quantitative real-time PCR was performed using an ABI 7500 Real-Time PCR system (Applied Biosystems). In a total volume of 15 *μ*l reaction mixture, 7.5 *μ*l cDNA templates were mixed with 7.5 *μ*l iTaq™ Universal SYBR Green Supermix (Bio-Rad Laboratories) and 0.45 *μ*l primers. The primers and probes used for the quantitative real-time PCR are shown in Table 1. All of the samples were determined in duplicate in three independent experiments.

### 2.8. Western Blot Analysis

The protein electrophoresis was performed using SDS-PAGE (polyacrylamide gel electrophoresis) for 90 min (condensed gel 10% and separate gel 5%). The volume of loading protein from each sample was 25 *μ*l. Then, protein was transferred to PVDF (polyvinylidene difluoride) membranes for 60 min and blocked in 5% skimmed milk for 1 hour. After washing at 80 r/min for 5 min, the membranes were placed together with primary antibodies in a shaker for 1°h and incubated overnight at 4°C. Then, the membranes were washed three times (shaked for 10 min each time) and incubated with HRP- (horseradish peroxidase-) conjugated fluorescence secondary antibody for 30 min at room temperature. After another three times of washing, immunoreactive proteins were detected by enhanced chemoluminescence (Odyssey Infrared Imaging System, USA). The target protein included Bax and Bcl-2, using *ß*-actin as the internal standard.

### 2.9. Statistical Analysis

Results were expressed as mean ± standard deviation (mean ± SD). Statistical analysis was performed with one-way ANOVA (one-way analysis of variance) using SPSS 17.0 software for Windows. *P* < 0.05 was considered to be statistically significant.

## 3. Results

### 3.1. Content Analysis of PN-F Decoction

The contents of mark ingredients in extracts of *Panax notoginseng* flower are listed in Table 2. The quality of the extract of *Panax notoginseng* flower was controllable.

### 3.2. Effects of PN-F Decoction on Quantification of Myocardial Infarct Size and Histopathological Analysis

**Figure 2:**
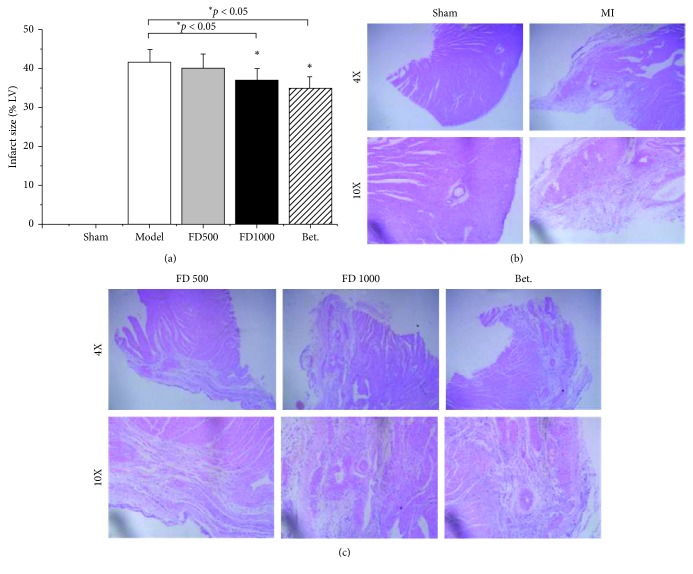
Effects of drugs on quantification of myocardial infarct size and histological morphology (a). After MI and administration, heart tissues were collected, subjected to HE staining, and pictured under the microscope of four or ten magnified fields (b). Sham: sham group; MI: myocardial infarction control group; FD500: *Panax notoginseng* flower decoction group (500 mg/kg/d); FD1000: *Panax notoginseng* flower decoction group (1000 mg/kg/d); Bet: Betaloc group (10 mg/kg/d). ^*∗*^*p*  <  0.05 and ^*∗∗*^*p* < 0.01 vs myocardial infarction control group.

As shown in Figure 2, compared to the MI group, quantification of myocardial infarct size in the Betaloc group and FD1000 group was significantly decreased (*p* < 0.05), indicating that PN-F significantly enhanced capillary density in the border area of MI.

Histological morphology of heart tissues was observed after HE staining (Figure 2). In the sham group, the myocardial fibers were arranged regularly with clear striations; no apparent degeneration or fibrosis was observed; the nucleus was clear, while infiltration of inflammatory cells was not obvious. In the MI control group, the histological sections showed a severe grade of fibrosis, unclear arrangement of striations, condensed sarcoplasm of the myocardium, infiltration of inflammatory cells, edema of the myocardial interstitium, dilation of vessels, and congestion. Compared to the MI control group, treatment with Betaloc and PN-F significantly decreased infarct size (inflammation, etc.); the symptoms were improved to some degree, indicating that drugs might be effective. However, there was no significant difference in mortality between the treatment and the MI control groups.

### 3.3. Effects of PN-F Decoction on MMVC

**Figure 3:**
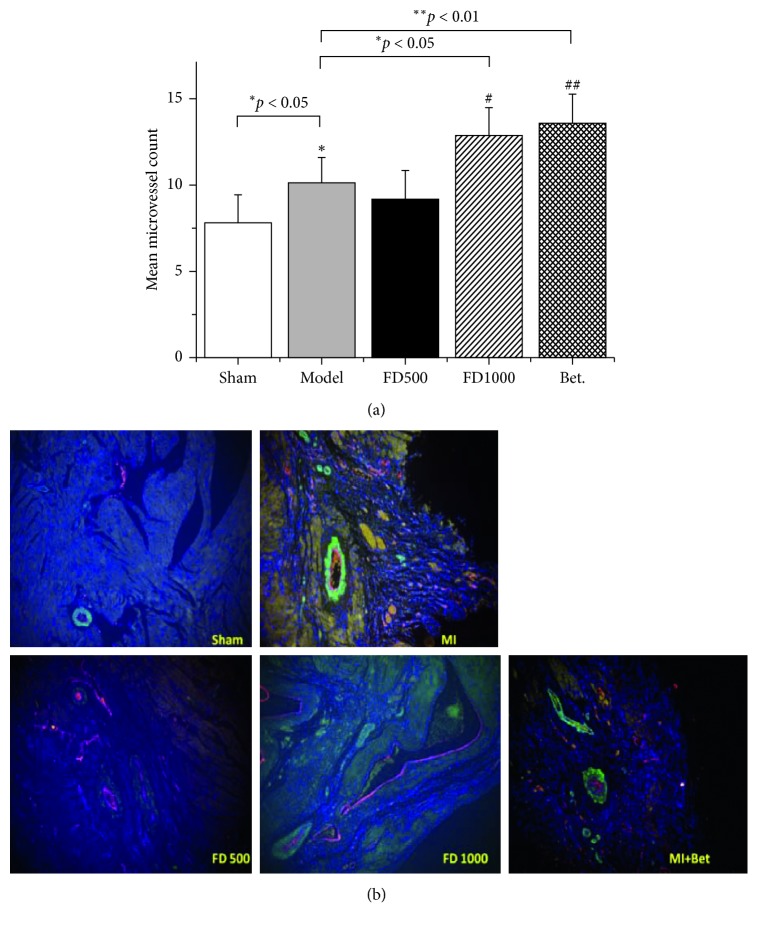
Effects of drugs on mean MVC (mean microvessel count) after 2 weeks of ischemia (a). After myocardial infarction and administration, heart tissues were collected and subjected to an immunofluorescent assay on SMA (green) and VWF (red) antibodies. Under the fluorescence microscope of 400 magnified fields, the minimal vessels were pictured and the numbers of vessels were counted (b). Sham: sham group; MI: MI control group; FD500: *Panax notoginseng* flower decoction (500 mg/kg/d); FD1000: *Panax notoginseng* flower decoction (1000 mg/kg/d); MI + Bet: Betaloc (10 mg/kg/d). Mean minimal vessel count is expressed as mean ± SD (*n*=5). ^*∗*^*p*  <  0.05 and ^*∗∗*^*p* < 0.01 vs sham group; ^#^*p* < 0.05 and ^##^*p* < 0.01 vs myocardial infarction control group.

Angiogenesis of minimal vessels in the infarct area could improve ischemia to some extent under the pathological condition of LAD artery ligation. In the study, minimal vessels in infarct zones were determined by an immunofluorescent assay after two weeks of ischemia. As shown in Figure 3, compared to the sham group, numbers of mean minimal vessels in MI control and treatment groups were significantly increased (*p* < 0.05), and the compensation did not appear in the sham group. Compared to the MI group, numbers of minimal vessels in the Betaloc group and FD1000 group were significantly increased (*p* < 0.05), indicating that PN-F might have angiogenesis effects on the infarcted area.

### 3.4. Effects of PN-F Decoction on Cell Apoptosis

**Figure 4:**
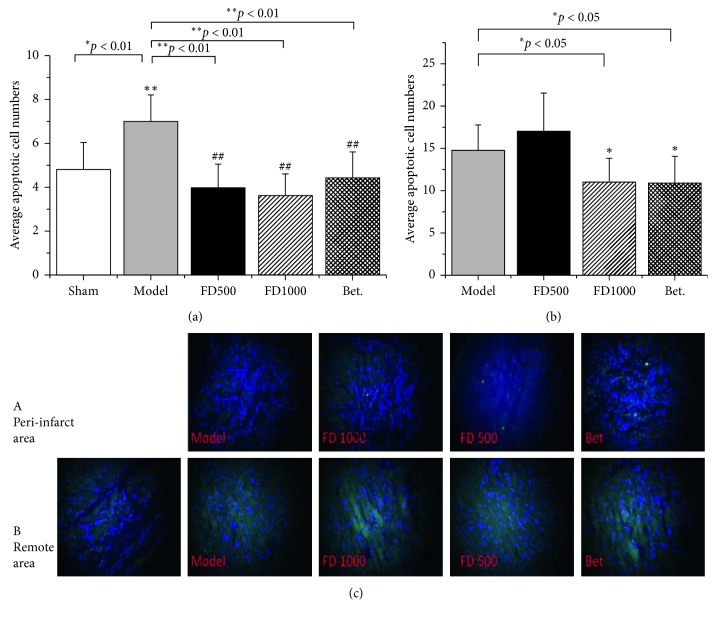
Effects of drugs on TUNEL data (apoptotic cells) in the peri-infarct and remote area after 2 weeks of ischemia. After myocardial infarction and administration, heart tissues were collected and subjected to a TUNEL assay. Under the fluorescence microscope of 200 magnified fields, apoptotic cells in the peri-infarct (a) and remote (b) area were pictured and counted (c). Sham: sham group; MI: myocardial infarction control group; FD500: *Panax notoginseng* flower decoction (500 mg/kg/d); FD1000: *Panax notoginseng* flower decoction (1000 mg/kg/d); MI + Bet: Betaloc (10 mg/kg/d). Apoptotic cell numbers are expressed as mean ± SD (*n*=5). ^*∗*^*p* < 0.05 and ^*∗∗*^*p* < 0.01 vs sham group; ^#^*p* < 0.05 and ^##^*p* < 0.01 vs MI control group.

Cell apoptosis was determined by TUNEL staining after myocardial ischemia (Figure 4). In the peri-infarcted area, there was no obvious apoptotic cell in the sham group, indicating no injury happened; however, in MI control and treatment groups, the number of apoptotic cells increased significantly, suggesting that LAD artery ligation could induce injury; furthermore, numbers in FD1000 and Betaloc groups were less than those in the MI control group (*p* < 0.05), indicating PN-F decoction might relieve injury induced by myocardial infarction.

In the remote area, apoptotic cells appeared in all groups, indicating LAD artery ligation might induce apoptosis in both the peri-infarct zone and remote area; the apoptotic cells in FD500, FD1000, and Betaloc groups were less than those in the MI control group (*p* < 0.05), indicating that PN-F decoction also inhibits apoptosis in the remote area.

### 3.5. Effects of PN-F Decoction on the Expressions of HIF-1, VEGFA, and KDR

**Figure 5:**
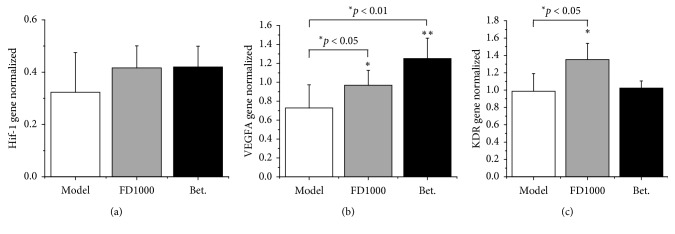
Effects of drugs on the gene expression in the MI area after 2 weeks of ischemia in rats. After myocardial infarction and administration, heart tissues were collected and subjected to qPCR. Sham: sham group; MI: myocardial infarction control group; FD1000: *Panax notoginseng* flower decoction (1000 mg/kg/d); MI + Bet: Betaloc (10 mg/kg/d). All experiments were performed at least in triplicate, and the results are expressed as mean ± SD. ^*∗*^*p* < 0.05 and ^*∗∗*^*p* < 0.01 vs sham control group; ^#^*p* < 0.05 and ^##^*p* < 0.01 vs MI control group.

The expressions of HIF-1, VEGFA, and KDR in the infarcted area were detected by qPCR after two weeks of ischemia. As shown in Figure 5, HIF-1 (hypoxia-inducible factor-1) expressions in the sham group were much less than those in MI control and drug groups, indicating no infarction happened leading to lack of HIF gene expression; expressions of HIF in FD1000 and Betaloc groups were higher than those in the MI control group, but the differences were not significant (*p* > 0.05), indicating that PN-F might induce production of HIF and VEGFA genes but not obviously.

For VEGFA (vascular endothelial growth factor-*α*) and KDR (kinase insert domain receptor), compared with the sham group, the expressions of the MI control group and FD1000 group obviously increased (*p* < 0.05), indicating the expressions of the FD1000 group were higher than those of the MI group, suggesting that PN-F could induce VEGFA and KDR expressions. Betaloc groups were higher than MI control groups in VEGFA (*p* < 0.01), but the differences were not significant in KDR (*p* > 0.05).

### 3.6. Effects of PN-F Decoction on the Bax and Bcl-2 Protein Expressions

**Figure 6:**
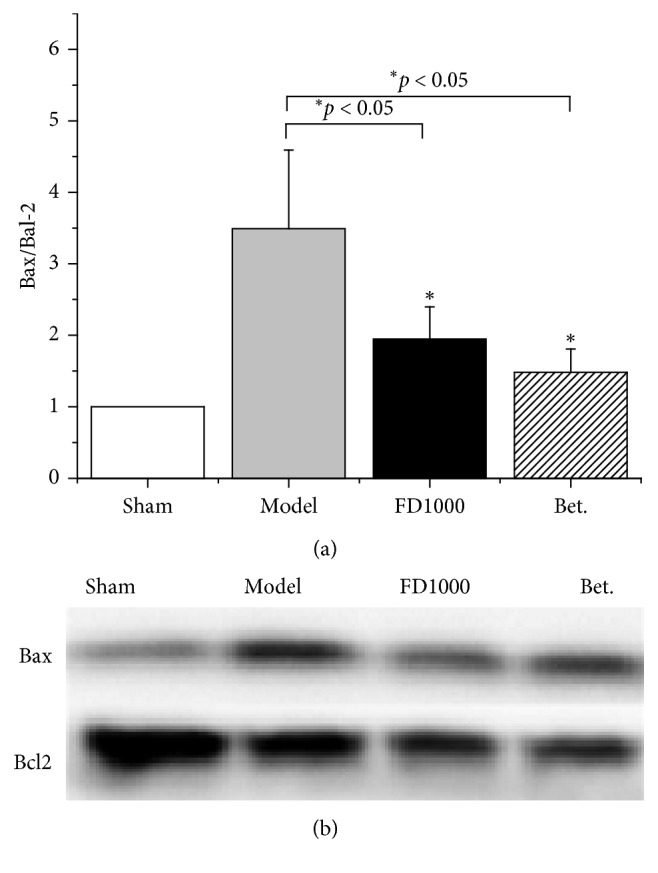
Effects of drugs on protein expression of Bax/Bcl-2 in the left ventricle after 2 weeks of ischemia. After myocardial infarction and administration, heart tissues were collected and subjected to western blotting. “Compared with the model group, the ratios of bax to bcl‐2 of FD1000 and Betaloc group were lower than the model group” (*p* < 0.05). Sham: sham group; MI: myocardial infarction control group; MI + FD 1000: *Panax notoginseng* flower decoction (1000 mg/kg/d); MI + Bet: Betaloc (10 mg/kg/d). All experiments were performed at least in triplicate, and the results are expressed as mean ± SD. ^*∗*^*p* < 0.05 and ^*∗∗*^*p* < 0.01 vs MI control group.

The expressions of the protein-infarcted area were detected after two weeks of ischemia. Results show (Figure 6) that, for ratio of Bax to Bcl-2, the FD1000 and Betaloc groups have ratio less than that of the MI control group (*p* < 0.05), indicating that PN-F might inhibit cell apoptosis in the infarcted area to protect heart tissue.

## 4. Discussions

The growth of new capillaries from current blood vessels, named angiogenesis, is a complex multistep process, comprising a series of cellular events that lead to neovascularization [22, 23]. Angiogenesis could also be an indicator of many diseases including wound healing, the menstrual cycle, cancer, various ischemic and inflammatory diseases, and even infarcted myocardium [24, 25]. Our previous study demonstrated that flowers of *Panax notoginseng* had proangiogenic effects on healthy and vessel-lost zebrafish which were damaged by VEGF receptor kinase inhibitor [26]. Intriguingly, our findings also indicated that purified saponin preparation from flowers of *Panax notoginseng* had more powerful and significant angiogenic/restorative effects than the extract from roots of *Panax notoginseng* [15, 17–19]. However, the potential angiogenesis effects of *Panax notoginseng* flowers (herb) on MI rats were unclear. Therefore, the present article paid attention to angiogenesis effects of flowers (herb) on MI rats.

There were various methods to establish an MI animal model, including drug induction, tube placement, and LAD ligation [27–30]. Among them, the most sophisticated method was LAD ligation. Unlike traditional LAD ligation, our experiment did not use ventilators. Although tracheal intubation could decrease probability of asphyxia, operation time might be extended and the possibility of animal death would increase. The key point of this operation was skill and speediness. The operation, including steps of thoracotomy, heart exposure, LAD ligation, heart reset, squeeze, and incision, should be over in a few minutes. Besides that, we should pay attention to the experiment temperature. It was very important to keep animal warm. Antibiotic prophylaxis was also necessary to avoid animal infectious. Results showed that the model was successfully established, and no special complications (including aneurysm formation and osseous and cartilaginous metaplasia formation) occurred after myocardial infarction. *β*-Blockers were clinically valuable for the setting of acute MI. Many evidences showed mortality was reduced when *ß*-blockers were administered early [31]. The use of oral *ß*-blockade was a class I indication according to clinical practice guidelines [32]. Therefore, the present study used *ß*-blockers as positive control medicine. In order to guarantee the repeatability of the experiment, the contents of chemical structures in *Panax notoginseng* flowers were determined using the HPLC method.

First, we need to observe morphologic changes after myocardial infarction. Myocardial infarct size was a strong predictor of cardiovascular events, so we observed histological morphology of heart tissues after HE staining. Results showed that the FD1000 group and Betaloc group significantly decreased infarct size, demonstrating PN-F had potential therapeutic functions on MI rats.

Second, in order to figure out its primary mechanism, minimal vessels in infarct zones were determined by an immunofluorescent assay after ischemia. Results showed that minimal vessels in the Betaloc group and FD1000 group were significantly increased, demonstrating that PN-F could improve minimal vessels in the infarct area. Apoptosis of cardiomyocytes also significantly contributed to loss of cardiac function after MI, as well as the development of heart failure [33, 34]. Therefore, cell apoptosis was determined by TUNEL staining after myocardial ischemia. Results showed that PN-F inhibits apoptosis in the peri-infarct zone and remote infarct area.

Next, we studied further molecular mechanism. Based on the above experiment, there were two prospects: growth of vessels and inhibition of cell apoptosis in the infarct area; thus, these two prospects were investigated.

The angiogenesis properties of VEGF during vascular development or tumor angiogenesis attracted people to evaluate its role in post-MI angiogenesis [35]. Hypoxia was a major stimulator of VEGF expression, which resulted from binding of HIF-1 to a hypoxia response element (HRE) [36]. HIF-1 could upregulate several genes to promote survival under low-oxygen conditions. HIF1-*α* was activated by hypoxia and then targeted a wide array of genes, including proangiogenesis genes (e.g., VEGF) [37]. Cardiac functions of mice could be improved after MI, which might relate to the increase of HIF1-*α* and VEGF expressions in cardiomyocytes [38]. VEGF included VEGFA and VEGF receptors (VEGFR-2 and kinase insert domain receptor (KDR)). Angiogenesis effects of VEGF resulted from VEGFR-2 activation. Interference of VEGF and KDR binding could inhibit angiogenesis and further suppress tumor growth [39, 40]. In the present study, results demonstrated that PN-F might induce expressions of HIF-1, VEGFA, and KDR. Therefore, it was possible that angiogenesis effects of PN-F might involve in upregulated expressions of HIF-1, VEGFA, and KDR genes.

On the other side, inhibition of cell apoptosis in the infarct area was also important. Both Bax and Bcl-2 protein related to apoptosis, but functions were different: Bcl-2 was specifically considered an important antiapoptotic protein [41], while Bax was proapoptotic protein [42]. The ratio of Bax to Bcl-2 could be a death signal of cells [43]. Our results showed that PN-F (herb) might inhibit cell apoptosis in the infarct area to protect heart tissue.

## 5. Conclusions

To sum up, our study revealed that PN-F (herb) could relieve symptoms of MI rats. Its mechanism might be to promote growth of minimal vessels in the infarct area through stimulation of HIF-1, VEGFA, and KDR and inhibit apoptosis of cardiomyocytes through downregulation of Bax/Bcl-2.

## Figures and Tables

**Table 1:** Information on primers.

ID^*∗*^	Primer	Primer sequence (5′ to 3′)	nmol	Purified method
NM_031836.2	VEGFA rat left	aaaaacgaaagcgcaagaaa	200	PAGE
	VEGFA rat right	Tttctccgctctgaacaagg	200	PAGE
NM_024359.1	Hypoxia-inducible factor-1 rat left	aagcactagacaaagctcacctg	200	PAGE
	Hypoxia-inducible factor-1 rat right	Ttgaccatatcgctgtccac	200	PAGE
NM_013062.1	KDR rat left	ggagattgaaagaaggaacgag	200	PAGE
	KDR rat right	Tggtacatttctggggtggt	200	PAGE

**Table 2:** The contents of mark ingredients in flower buds of *Panax notoginseng* (*µ*g/g).

Rg1	Rb1	Re	R1	Rb3	Fc	Ft1	Rg3-20s	Rg3-20R	Rh1	Rd	Rf	Rb2	Rh2-20s
6.30	841.32	0.08	0.39	798.30	144.99	0.33	106.64	7.8	3.94	2032.54	23.14	31510.88	50809.85

## Data Availability

The data used to support the findings of this study are available from the corresponding author upon request.

## Abbreviations

ANOVA: Analysis of variance
BCA: Bicinchoninic acid
Bet: Betaloc
CPR: Cardiopulmonary resuscitation
DAPI: Diamidino phenylindole
FD: *Panax notoginseng* flower decoction
HE: Hematoxylin and eosin
HIF-1: Hypoxia-inducible factor-1
HRP: Horseradish peroxidase
KDR: KDR (kinase insert domain receptor)
LAD: Left anterior descending
MI: Myocardial infarction
MMVC: Mean minimal vessel count
PBS: Phosphate-buffered saline
PN-F: *Panax notoginseng* flower
PMSF: Phenyl methane sulfonyl fluoride
SD: Standard deviation
SDS-PAGE: SDS-polyacrylamide gel electrophoresis
TUNEL: Terminal deoxynucleotidyl transferase-mediated dUTP nick-end labeling
VEGFA: Vascular endothelial growth factor-*α*
VWF: von Willebrand factor
*α*-SMA: *α*-Smooth Muscle Actin.
